# Chemical modification utilizing a terminal structure exposed on the specific surface of polymer-metal complex nanocrystals†

**DOI:** 10.1039/c9ra10244b

**Published:** 2020-02-07

**Authors:** Ryuju Suzuki, Tsunenobu Onodera, Hitoshi Kasai, Hidetoshi Oikawa

**Affiliations:** Institute of Multidisciplinary Research for Advanced Materials, Tohoku University Katahira 2-1-1, Aoba-ku Sendai 980-8577 Japan ryuju.suzuki.e6@tohoku.ac.jp +81-22-217-5587

## Abstract

It has been difficult to selectively modify the surface of molecular crystals by chemical reactions because they usually have no reaction points on their surfaces. In this paper, focusing on the unique nanocrystal surface of the polymer metal complex (PMC) [{Cu_2_(μ-Br)_2_(PPh_3_)_2_}(μ-bpy)]_*n*_ having an exposed reactive terminal chain, we successfully modified the surface of PMC nanocrystals (NCs) through an alkylation reaction. Interestingly, after the alkylation reaction, the luminescence spectrum of PMC NCs blue-shifted, and the luminescence quantum yield increased. PMC NCs with a large specific surface area showed optically peculiar or characteristic properties compared with the corresponding bulk crystals. PMC NCs have high potential as a new class of luminescent materials due to their surface effect.

## Abstract 2

We modified the surface of polymer metal complex nanocrystals (PMC NCs) with the alkylation reaction by utilizing coordinatively unsaturated bipyridine ligands exposed on the (010), and successfully changed the luminescence properties of PMC NCs.

## Graphical abstract

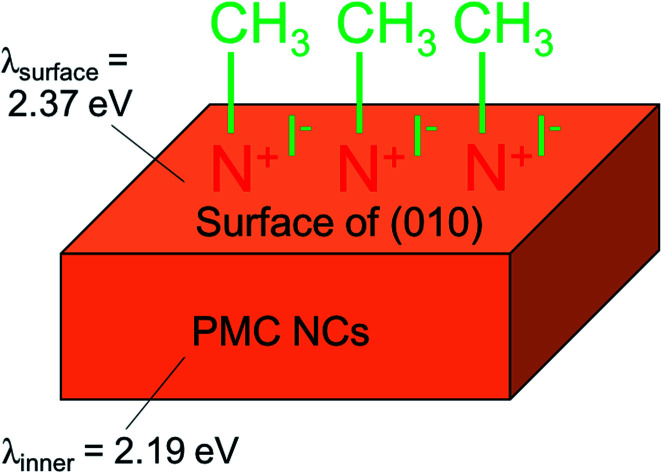

## 1. Introduction

Polymer metal complexes (PMCs) formulated as [{Cu_2_(μ-Br)_2_(PPh_3_)_2_}(μ-L)]_*n*_ (L denotes the ligand of bipyridine derivatives) have unique optical properties derived from metal-to-ligand charge transfer (MLCT).^1^ In particular, the luminescence energy strongly depends on the π* level of bipyridine ligands, and the luminescence peak position can be controlled by changing the other bipyridine derivatives. Interestingly, their unique optical properties are observed in the solid state.

In our previous studies, we have successfully fabricated PMC nanocrystals (NCs) by the heterogeneous reaction process, that is, a nanocrystallization method already established by us^2–6^ and observed the size effect on their optical properties.^2^ In addition, we found that these PMC NCs have a unique crystal lattice and surface structure on the basis of the crystallographical study of PMCs. The polymeric chains extend perpendicular to the (010) plane in PMC NCs, thereby exposing the terminal chains of PMCs. So, we considered that it is possible to perform surface modification using coordinatively unsaturated ligands of the terminal chains to modulate and/or tune the unique physicochemical properties. We previously revealed that coordinatively unsaturated bipyridine ligands would be exposed on the (010) surface of PMC NCs by adsorbing the suitably selected metal nanoparticles.^3^

Total surface modification for molecular crystals can be performed by the physisorption process^7–10^ because the surface of the molecular crystals has often no reaction points to chemically react with modifiers. In contrast, PMC NCs having unique surface crystal structures can be modified by chemical bonding even though they are molecular crystals.

In this paper, we focused on the alkylation reaction as a method for the surface modification of PMC NCs. The alkylation reaction of tertiary amines including N-heteroaromatic rings with alkyl halide is likely to proceed under moderate conditions without forming any byproducts,^11–13^ and the bipyridine ligands exposed on PMC NCs can also react with alkyl halide as a modifier. Herein, we have reported the surface effect of PMC NCs through the analysis of their structure and optical properties.

## 2. Result and discussion

**Scheme 1:**
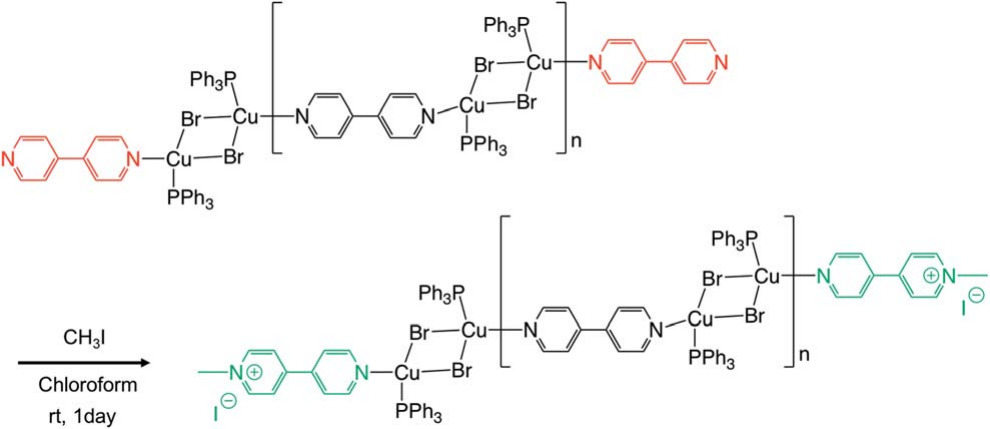
Alkylation reaction of PMC (polymer metal complex) with methyl iodide.

**Fig. 1:**
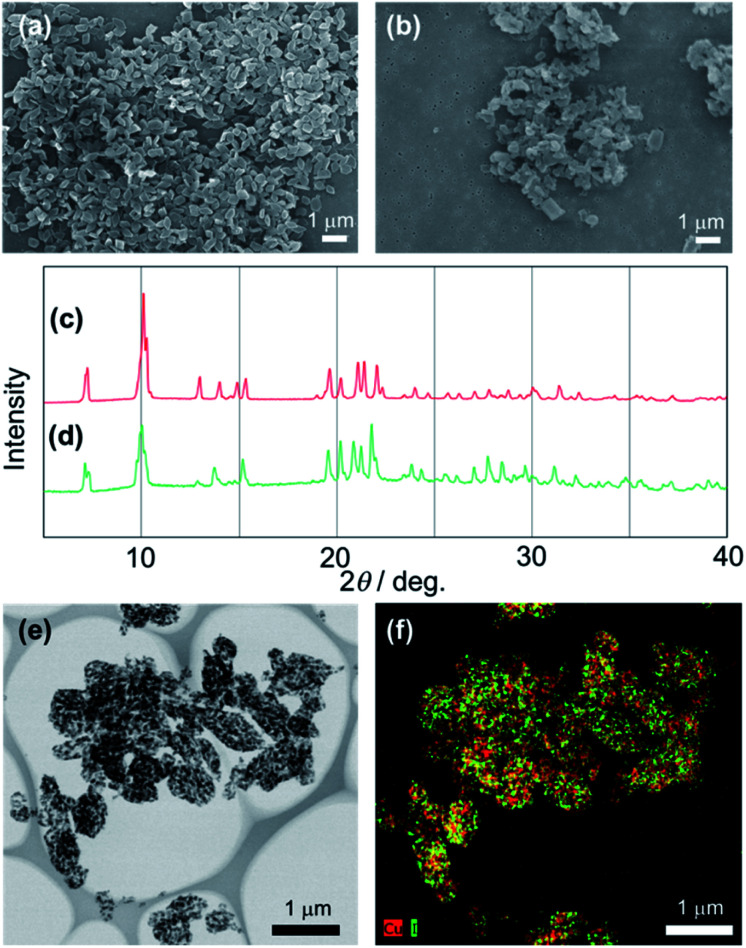
SEM images of PMC NCs reacted (a) without and (b) with methyl iodide. Powder XRD patterns of PMC NCs reacted (c) without and (d) with methyl iodide. (e) TEM image and (f) the corresponding EDS mapping of PMC NCs reacted with methyl iodide. In the EDS mapping, red and green points indicate Cu atoms of PMC and iodine atoms of methyl iodide, respectively.

We performed the alkylation reaction of PMC NCs with methyl iodide, as shown in Scheme 1. The reaction proceeded in a chloroform-suspension state of PMC NCs. The morphology of PMC NCs was a parallelogram-like plate, and it hardly changed before and after the alkylation reaction, as shown by the SEM images (Fig. 1(a) and (b)). This fact was also confirmed by DLS measurements, and the average size of PMC was maintained after the alkylation reaction. In addition, the powder XRD patterns of PMC NCs were also maintained after the reaction (Fig. 1(c) and (d)). On the other hand, the TEM image and EDS mapping of PMC NCs revealed that the iodide atoms introduced from methyl iodide were located with Cu atoms inside the PMC NCs (Fig. 1(e) and (f)). These results suggested that methyl iodide reacted with bipyridine ligands exposed on the surface of PMC NCs without affecting the crystal structure.

**Fig. 2:**
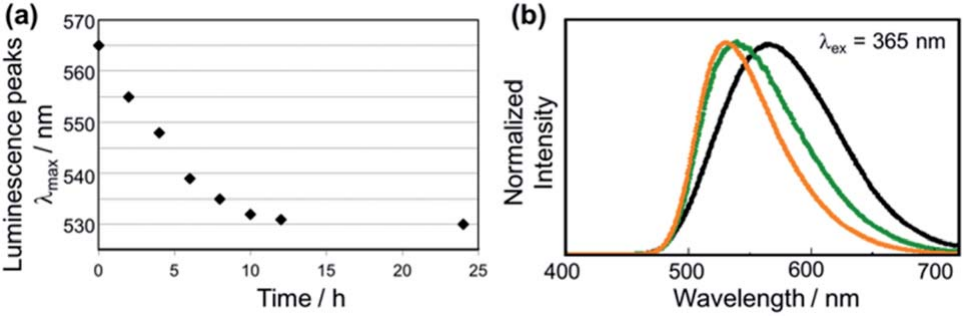
(a) Changes in luminescence peak during the alkylation reaction time of PMC NCs with methyl iodide. (b) Luminescence spectra of PMC; (black) without the alkylation reaction (bulk crystal and nanocrystals have the same spectrum), (green) bulk crystal and (orange) nanocrystals reacted with methyl iodide.

Fig. 2(a) shows the investigation of the alkylation reaction process by monitoring the wavelength of the luminescence peak. The luminescence wavelength was initially *λ*_em_ = 565 nm and gradually blue-shifted with the reaction time. Finally, the reaction would be completed at *λ*_em_ = 530 nm after 24 hours, and the final luminescence spectrum of PMC NCs is indicated by an orange line, as shown in Fig. 2(b). In addition, we performed the same reaction using a PMC bulk crystal, and the luminescence spectrum after the completion of the reaction is shown by a green line in Fig. 2(b). After the reaction, the luminescence spectrum of PMC NCs slightly blue-shifted compared to that of the bulk crystal.

**Fig. 3:**
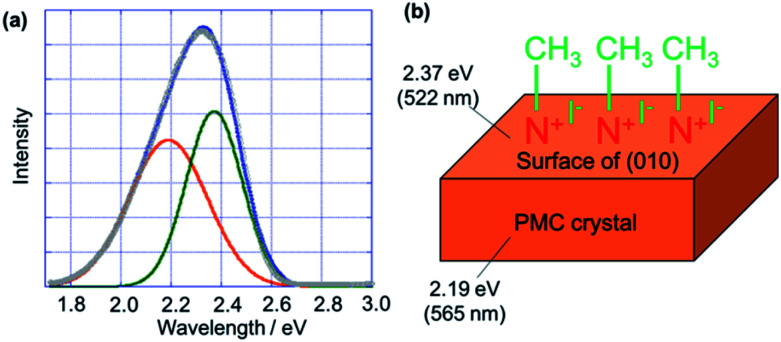
(a) Peak fitting of luminescence spectra (gray) of PMC NCs reacted with methyl iodide, (blue) whole fitted spectrum having (orange and green) two components. (b) Structural image of surface-modified PMC NCs with methyl iodide. A short wavelength component of fitted spectrum (*λ* = 2.37 eV) was derived from the surface of PMC NCs, and their inner crystal maintained the luminescence spectrum of non-alkylation-reacted PMC NCs (*λ* = 2.19 eV).

We simulated the luminescence spectrum of PMC NCs after the alkylation reaction by dividing it into two components (Fig. 3(a)) to reveal the blue-shift of the luminescence spectrum. We assumed that the crystal after the reaction would be composed of two domains, namely, the crystal surface that reacted with methyl iodide and the unchanged inner crystal structure (Fig. 3(b)). Actually, the luminescence spectrum was well-fitted with two peaks. One peak appeared at *λ*_em_ = 2.19 eV (565 nm), which nicely agreed with the spectrum of non-alkylation-reacted PMC NCs. On the other hand, the other peak appeared at *λ*_em_ = 2.37 eV (522 nm), which would be derived from the reacted crystal surface. The luminescence properties of the PMC derivatives were assigned to metal-to-ligand charge transfer (MLCT).^1^ This means that the luminescence energy strongly depends on the N-heteroaromatic ligands.^1^ In our case, we considered that the π* energy level of bipyridine ligands as the N-heteroaromatic ligands on the surface of the nanocrystal was destabilized, which led to the appearance of a new peak at *λ*_em_ = 2.37 eV (522 nm). In addition, the luminescence intensity of the new peak was higher than that of the peak derived from the unchanged inner crystal structure. As a result, we suggested that the luminescence spectrum of PMC NCs after the alkylation reaction blue-shifted. This would explain the reason for the larger luminescence changes in PMC NCs compared to that in the PMC bulk crystal. In general, nanocrystals have a large proportion of molecules on the surface of the whole crystal compared to the bulk crystal. So, the proportion of reacted bipyridine ligands in PMC NCs is larger than that in the PMC bulk crystal. Accordingly, the luminescence peak of PMC NCs after the alkylation reaction blue-shifted compared to that of the PMC bulk crystal. This fact could also support that the alkylation reaction proceeded on the surface of the PMC crystal.

Interestingly, the luminescence quantum yield of PMC NCs increased from 15% to 40% through the alkylation reaction and similarly, that of the bulk crystal increased from 40% to 50%. As we have already reported, the (010) plane of the PMC crystal is a reactive surface, on which the bipyridine ligands are exposed.^3^ The bipyridine ligands as terminal chains would easily adsorb molecules and ions in the surrounding environment. So, the luminescence was quenched by the adsorbed molecules, especially oxygen, on the surface of PMC NCs. On the contrary, the luminescence would not be quenched in PMC NCs that reacted with methyl iodide because the (010) plane as a reactive surface was protected by methyl iodide. Otherwise, it is conceivable that the molecular vibration of PMC is suppressed by the reaction with methyl iodide on the crystal surface. This means that the non-radiative transition would be inhibited. In any case, the luminescence quantum yield of PMC NCs was successfully improved to the same level as that of non-alkylation-reacted PMC bulk crystals.

**Fig. 4:**
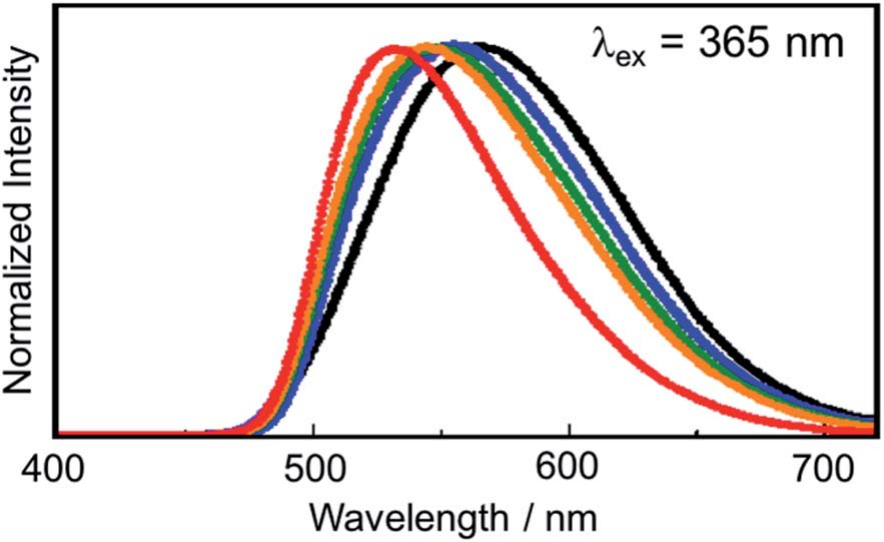
Luminescence spectra of PMC NCs reacted with (red) methyl iodide, (orange) ethyl iodide, (green) butyl iodide and (blue) octyl iodide. The black solid line is the luminescence spectrum of PMC NCs without the alkylation reaction.

We also investigated the alkylation reaction using other kinds of alkyl iodides having different alkyl chain lengths (Fig. 4). It was revealed that the luminescence spectra of PMC NCs blue-shifted as the alkyl chain length decreased. In other words, PMC NCs reacting with methyl iodide provided the most blue-shifted luminescence spectrum because other bulky long alkyl chains would prevent surface modification. Obviously, PMC NCs reacting with methyl iodide also exhibited the highest luminescence quantum yields (Table S1†) due to the high surface coverage. Even at different coverages among alkyl iodides, these results indicated that surface modification is possible with the alkylation reaction as well as with CH_3_I. The proceeding of the alkylation reaction could be confirmed by the change in luminescence properties, which is a point of advantage in our surface modification method. We think that this phenomenon is achieved only by chemical bonding and not by physisorption. In fact, we tried to modify PMC NCs with some metal nanoparticles (*e.g.*, Au, Ag); the luminescence spectrum did not change in spite of sufficient adsorption. To control the luminescence spectrum of PMC NCs by surface modification, it is necessary to bind the modifier directly to bipyridine ligands exposed on the crystal surface by chemical bonding and change the π* energy level of the bipyridine ligands.

## 3. Conclusions

We successfully modified the surface of [{Cu_2_(μ-Br)_2_(PPh_3_)_2_}(μ-bpy)]_*n*_ nanocrystals (PMC NCs) with the alkylation reaction by skilfully utilizing coordinatively unsaturated bipyridine ligands exposed on the (010) surface. In other words, alkyl iodides can recognize and react with bipyridine ligands exposed on the crystal surface without decomposing the crystal structure. After the reaction, the luminescence spectrum of PMC NCs blue-shifted, and the luminescence quantum yield increased. The analysis of the luminescence spectrum suggested that the novel optical properties were derived from changing the energy level of the PMC molecules on the crystal surface. Until now, it has been difficult to modify the surface of organic nanocrystals by chemical bonding, but we believe that our strategy has found the possibility of chemically modifying the surface by controlling the crystal structure of nanocrystals. Molecules or atoms on solid surfaces often exhibit curious properties^14–17^ and so, the surface modification based on chemical bonds may lead to the development of novel properties, which cannot be achieved by physical adsorption. Of course, our material design can be applied in various fields other than luminescence properties. PMC NCs represent a new class of materials because of their unique surface effect and have great potential to reveal the novel surface effect of molecular crystals. We are now trying to control the luminescence properties of PMC NCs by using alkyl iodides possessing functional molecules.

## 4. Experimental section

### Materials and preparations

Alkyl iodide derivatives and all solvents were commercially available. [{Cu_2_(μ-Br)_2_(PPh_3_)_2_}(μ-bpy)]_*n*_ nanocrystals (PMC NCs) were prepared by the already-established heterogeneous reaction process,^2–6^ and the color of the resulting PMC NCs was yellow in a powder state. On the other hand, the bulk crystal was prepared according to the procedure described elsewhere.^1^

### Fabrication of PMC NCs

PMC NCs were fabricated by the already-established heterogeneous reaction process.^2^ First, 200 μL of acetone solution of PPh_3_ (0.5 mg, 2 μmol) was injected into vigorously stirred 10 mL of aqueous solution of 4,4′-bipyridine (0.6 mg, 4 μmol), so that PPh_3_ nanocrystals were formed and dispersed in a 4,4′-bipyridine aqueous solution. Subsequently, 200 μL of acetonitrile solution of CuBr (0.3 mg, 2 μmol) was added dropwise into the above dispersion liquid. The color of the dispersion liquid changed from white to pale yellow. The obtained pale yellow NCs were filtered, washed with acetone, and re-dispersed in distilled water. PMC NCs could be almost quantitatively obtained over 90% yields.

### Modification on the surface of PMC NCs through the alkylation reaction

The powder of PMC NCs (20 mg, 0.02 mmol) was dispersed in chloroform and then, methyl iodide (3 μL, 0.03 mmol) was added to the NC suspension. The mixture was stirred at room temperature for 24 h; subsequently, the suspension was filtrated and washed with acetonitrile and acetone. Consequently, surface-modified PMC NCs were obtained as a yellow-green powder. Similarly, the PMC bulk crystal was reacted with methyl iodide.

### Measurements for characterization

Structural and morphological characterizations for the synthesized PMC NCs were performed by scanning electron microscopy (SEM; JSM-6700F, JEOL) and transmission electron microscopy (TEM, Titan 80-300, FEI, operated at 300 kV). The electron beam diffraction pattern was also obtained by TEM (Titan 80-300, FEI, operated at 300 kV). EDS analysis was conducted using TEM (Titan 3 G2 60-300, FEI, operated at 60 kV). Luminescence spectrum was measured with a luminescence spectrometer (F-7000, Hitachi), and the luminescence quantum yields were determined by a total fluorescence spectrometer equipped with an integrating sphere (IZ-CT-25TP, Bunkoukeiki).

## Conflicts of interest

There are no conflicts to declare.

## Supplementary Material

RA-010-C9RA10244B-s001
