# Supplementary material for: Chemical modification utilizing a terminal structure exposed on the specific surface of polymer-metal complex nanocrystals
Source: RSC Adv. 2020 Feb 7;10(11):6135–8. doi: 10.1039/c9ra10244b (PMC9049687; doi:10.1039/c9ra10244b)
Supplement: RA-010-C9RA10244B-s001 [file RA-010-C9RA10244B-s001.pdf]

Electronic Supplementary Material (ESI) for RSC Advances.
This journal is © The Royal Society of Chemistry 2020

Supplementary information

Table S1. Luminescence quantum yields (Φ) of PMC NCs reacted with alkyliodides having different alkyl chain length.

alkyliodides	Luminescence quantum yields of PMC NCs (Φ)
methyl iodide	40%
ethyl iodide	24%
butyl iodide	18%
octyl iodide	15%
